# Supplementary material for: Effects of nonlinearity on Anderson localization of surface gravity waves
Source: Nat Commun. 2024 Jul 8;15:5726. doi: 10.1038/s41467-024-49575-5 (PMC11231258; doi:10.1038/s41467-024-49575-5)
Supplement: Supplementary file 1 — Supplementary Information [file 41467_2024_49575_MOESM1_ESM.pdf]

Supplementary Information for "Effects of nonlinearity on Anderson localization of surface gravity waves"

Guillaume Ricard, Filip Novkoski, and Eric Falcon
Université Paris Cité, CNRS, MSC, UMR 7057, F-75013 Paris, France

In this Supplementary Information, we present details on the experimental setup (Sect. S1), on the spatial evolution of the surface elevation η in different cases (Sect. S2 A), on numerical simulations of the spatiotemporal spectrum (Sect. S2 B), on a comparison between random and periodic bottoms (Sect. S2 C), on the matrix transfer method (Sect. S2 D), on viscous dissipation along the canal (Sect. S2 E) and on the variation of the characteristic lengths with the frequency (Sect. S2 F). Supplementary references are in (Sect. S3). Notations as in the aforementioned text.

S1. SUPPLEMENTARY METHODS

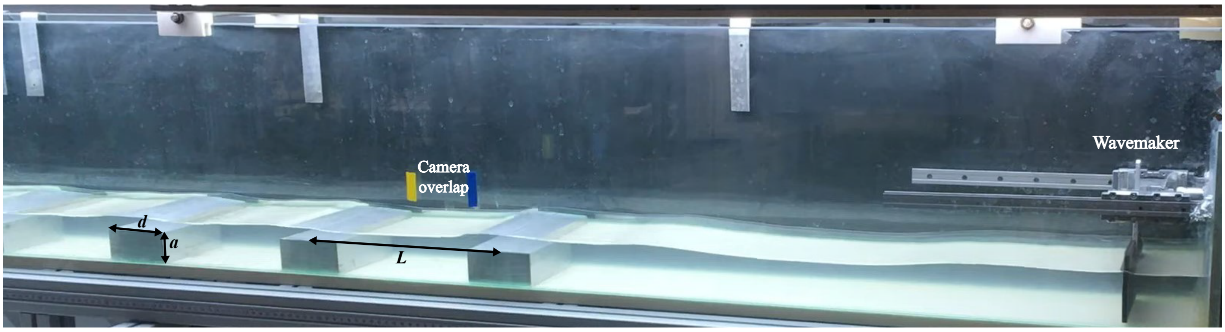

Figure S1: **Photography of the experimental setup.** The wavemaker and part of the periodic bathymetry are visible (the first 1.2 m of the canal). L = 25 cm, a = 4 cm, d = 8 cm, fluid depth h = 5.5 cm. A video showing gravity surface waves halted by the periodic bathymetry is available in Supplementary Information Video 1.

S2. SUPPLEMENTARY DISCUSSION

A. Spatial evolution of the surface elevation

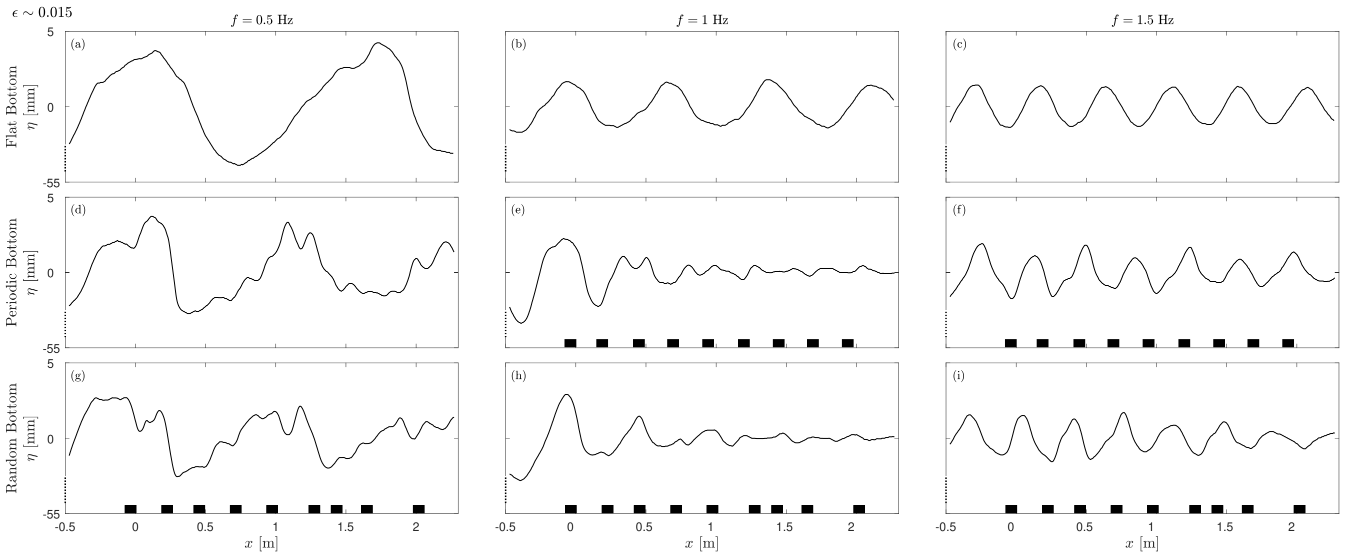

Figure S2: **Spatial evolution of the surface elevation** $\eta(x)$ **along the first 2.8 m of the canal** for a fixed time, wave steepness, $\epsilon \sim 0.015$, and different frequencies, $f =$ (a) 0.5, (b) 1, (c) 1.5 Hz, for a flat bottom. (d), (e), and (f) same for periodic bathymetry. (g), (h), and (i) same for random bathymetry. The wavemaker is located on the left-hand side. Black rectangles indicate the location of bars in the canal. A video showing the corresponding spatiotemporal evolution of $\eta(x,t)$ is available as Video 2 in the Supplementary Information.

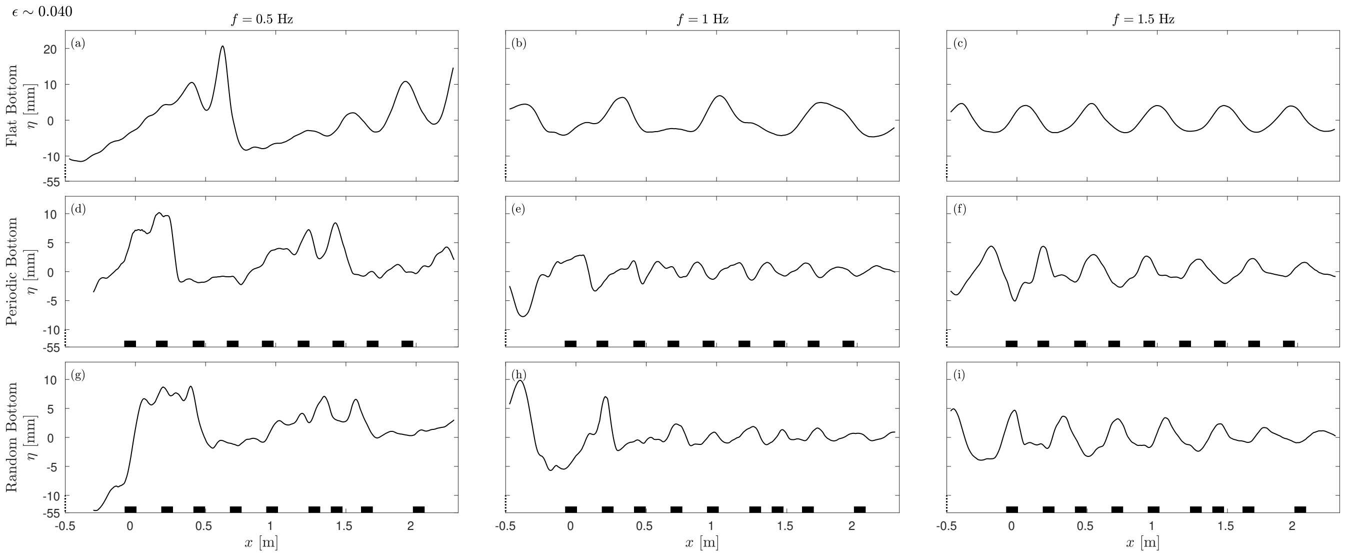

Figure S3: **Spatial evolution of the surface elevation** $\eta(x)$ **along the first 2.8 m of the canal** for a fixed time, wave steepness, $\epsilon \sim 0.040$, and different frequencies, $f =$ (a) 0.5, (b) 1, (c) 1.5 Hz, for a flat bottom. (d), (e), and (f) same for periodic bathymetry. (g), (h), and (i) same for random bathymetry. The wavemaker is located on the left-hand side. Black rectangles indicate the location of bars in the canal. A video showing the corresponding spatiotemporal evolution of $\eta(x,t)$ is available in Supplementary Information Video 3.

B. Numerical simulations of the spatiotemporal spectrum

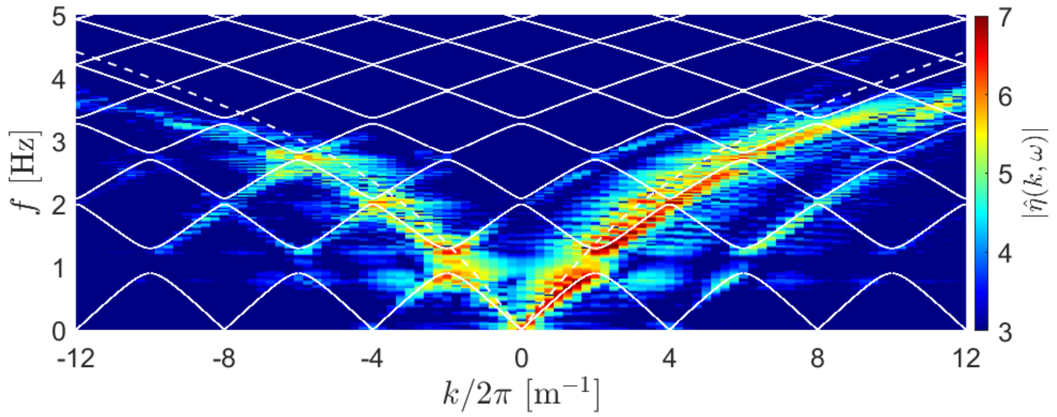

Figure S4: **Numerical simulations of the spatiotemporal spectrum of the surface elevation, $|\hat{\eta}(k,\omega)|$.** The case shown here is analog to the experiment of Fig. 2c: sweep forcing $f \in [0.5, 1.4]$ Hz with a weak steepness $\epsilon = 0.015$ for a periodic bottom. Dashed-white lines: Eq. (1b). Solid-white lines: Eq. (4) with a $1/L$ spatial periodicity. Logarithmic-scale colorbar.

C. Comparison between the random and periodic cases

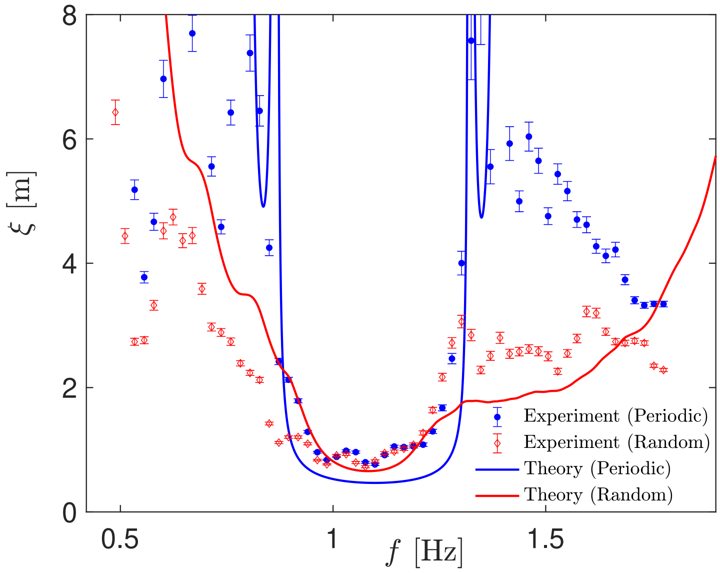

Figure S5: **Localization length ξ as a function of the wave frequency f for the random (red) and periodic (blue) cases.** Symbols correspond to the experiments for $\epsilon = 0.015$ and solid lines to the linear theory from Eq. (4). Error bars are related to the exponential fit error (see Fig. 3).

D. Theoretical localization length

We consider a wave $\eta_I(x) = A_I e^{ikx} + A_{IR} e^{-ikx}$ before the lattice of steps, with $A_I = 1$ its normalized incident amplitude and A_{IR} its reflected. After the lattice of steps, the transmitted wave reads $\eta_T = A_T e^{ikx} + A_{TR} e^{-ikx}$ with $A_{TR} = 0$ (no reflection is coming from the beach). Potential equations of linear gravity water waves propagating and scattering over topographical bottoms can be reduced to $\nabla \left[\frac{1}{k_i^2} \nabla \eta(x) \right] + \eta(x) = 0$ with $\nabla \equiv \partial/\partial x$ and k_i satisfying Eq. (1) [1, 2]. The boundary conditions at each step (i.e., across depth variations from h to h_1) being continuous, the wave amplitude η and the quantity $\frac{1}{k_i^2} \nabla \eta$ are continuous across each step [1–3], leading to the relationship between the incident wave amplitude before the lattice and the transmitted amplitude after the lattice by matrices as [2]

$$\begin{pmatrix} A_I = 1 \\ A_{IR} \end{pmatrix} = \mathcal{T} \begin{pmatrix} A_T \\ A_{TR} = 0 \end{pmatrix} = \begin{pmatrix} \mathcal{T}_{11} & \mathcal{T}_{12} \\ \mathcal{T}_{21} & \mathcal{T}_{22} \end{pmatrix} \begin{pmatrix} A_T \\ 0 \end{pmatrix}, \tag{s1}$$

with $\mathcal{T}$ the transfer matrix that depends on k, k_1, the incident wave frequency, the periodic or random positions of the steps, and on their width d. $\mathcal{T} = \prod_{p=1}^{N} \mathcal{T}_p$ is the transfer matrix defined as the product of the transfer matrices of every p-th step $\mathcal{T}_p$, that reads [2]

$$\begin{aligned} \mathcal{T}_p =& \frac{1}{4} \begin{pmatrix} (1+G)e^{i(k_1-k)x_p} & (1-G)e^{-i(k_1+k)x_p} \\ (1-G)e^{i(k_1+k)x_p} & (1+G)e^{-i(k_1-k)x_p} \end{pmatrix} \\ & \times \begin{pmatrix} (1+1/G)e^{i(k-k_1)(x_p+d)} & (1-1/G)e^{-i(k+k_1)(x_p+d)} \\ (1-1/G)e^{i(k+k_1)(x_p+d)} & (1+1/G)e^{-i(k-k_1)(x_p+d)} \end{pmatrix}, \end{aligned} \tag{s2}$$

with $G = k/k_1$ and x_p the location of the beginning of the p-th step. Note that the locations x_p can be periodic or random.

To demonstrate a change of the wave amplitude along the lattice, the localization length ξ is defined as the characteristic length of an exponential decrease of the wave amplitude [4]

$$\eta(x) = \eta_0 \exp(-x/\xi) = T\eta_0, \tag{s3}$$

with $T = \eta/\eta_0 = 1/\mathcal{T}_{11}$ the transmission coefficient, with $\mathcal{T}_{ij}$ the coefficient of the i-th row and j-th column of $\mathcal{T}$, and $\eta_0 \equiv \eta(x=0)$. From Eq. (s3), the localization length thus reads

$$\xi(\omega) = -\frac{NL}{\ln(T)} = \frac{NL}{\ln(\mathcal{T}_{11})}, \tag{s4}$$

with N the number of steps, L the averaged space between two consecutive steps, and NL the total lattice length.

E. Theoretical viscous dissipation along the canal

For the flat bottom, dissipative effects are estimated using the dissipation length l_d defined as

$$\eta = \eta_0 \exp(-x/l_d). \tag{s5}$$

Considering that the damping is mostly due to viscous dissipation by a fully contaminated surface, and by friction on the walls and at the bottom [5], l_d reads

$$\begin{aligned} l_d =& \mathcal{U} / \left[\frac{k\sqrt{\nu\omega}}{2\sqrt{2}} + \sqrt{\frac{\nu\omega}{2}} \left(\frac{k}{\sinh(2kh)} + \frac{1}{L_y} \right) \right], \\ \mathcal{U} =& \frac{1}{2} \sqrt{\frac{g}{k\tanh(kh)}} \left[\tanh(kh) + \frac{kh}{\cosh^2(kh)} \right], \end{aligned} \tag{s6}$$

with $\mathcal{U}$ the group wave velocity. In the range of probed frequencies $f \in [0.48, 2]$ Hz and $h = 5.5$ cm, the theoretical dissipation length is $l_d \in [9.2, 35]$ m (see Fig. S6), which is much larger than the canal length $L_x = 4$ m, and agrees with the experimental estimation $l_d \sim 20 \pm 10$ m. Note that using a depth $h_1 = 1.5$ cm leads to a dissipation length ~ 4.5 m also larger than the canal length. Viscous dissipation is hence negligible for the present study.

F. Characteristic lengths along the canal

To observe Anderson localization and Bragg reflection, it is required to respect $L \ll \lambda \ll \xi \ll l_d$, and ξ has not to be too large compared to L_x. The variations of these lengths are plotted in Fig. S6. This theoretical plot shows that the inequality is well respected everywhere in the frequency range of interest (vertical dashed lines) but for $f \sim 1$ Hz where $\lambda \approx \xi_r$ (random case) and for $0.94 < f < 1.2$ Hz where $\lambda > \xi_p$ (periodic case). This explains why the experimental values of ξ are not as small as they should be for $0.94 < f < 1.2$ Hz in the case of a periodic bottom [Fig. 4a]. This might also prevent fully exploring the nonlinear effects in this range for $0.94 < f < 1.2$ Hz in the periodic case.

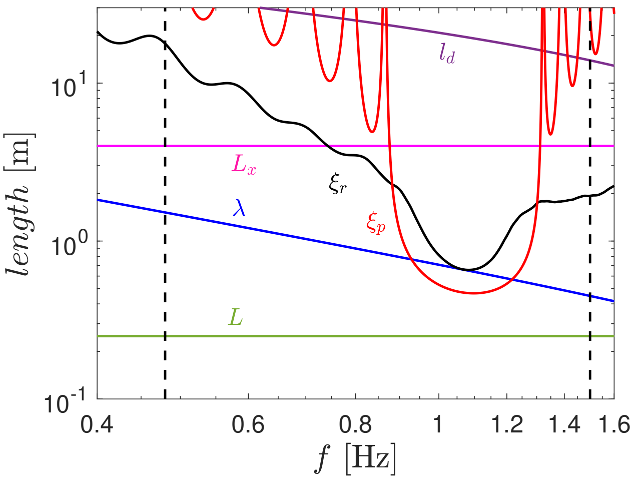

Figure S6: **Evolution of the theoretical lengths of the problem versus the incident wave frequency f.** One finds $L \lesssim \lambda \lesssim \xi < L_x \ll l_d$ as expected for waves to be localized, with $L = 0.25$ m the disorder scale, λ the wavelength from Eq. (1b), ξ the localization length from Eq. (s4), $L_x = 4$ m the system size, and l_d the dissipative length from Eq. (s6) of Section S2 E. ξ_r is the localization length for the random case, and ξ_p is the one for the periodic case. Note that in the band gap near $f \sim 1$ Hz, one has $\lambda \gtrsim \xi_p$ (periodic case), explaining why the experimental value of ξ for the periodic case is slightly higher than the predicted one near 1 Hz [see Fig. 4a]. The vertical dashed lines correspond to the experimental frequency range of interest.

S3. SUPPLEMENTARY REFERENCES

[1] Z. Ye, Water wave propagation and scattering over topographical bottoms, Phys. Rev. E **67**, 036623 (2003)

[2] M.-J. Huang, C.-H. Kuo, and Z. Ye, Gravity waves over topographical bottoms: Comparison with experiment, Phys. Rev. E **71**, 011201 (2005)

[3] Y. Zhang, Y. Li, H. Shao, Y. Zhong, S. Zhang, and Z. Zhao, Band gaps and localization of surface water waves over large-scale sand waves with random fluctuations, Phys. Rev. E **85**, 066319 (2012)

[4] P.-G. Luan and Z. Ye, Acoustic wave propagation in a one-dimensional layered system, Phys. Rev. E **63**, 066611 (2001)

[5] P. Devillard, F. Dunlop, and B. Souillard, Localization of gravity waves on a channel with a random bottom, J. Fluid Mech. **186**, 521 (1988)
