# Supplementary material for: Effects of nonlinearity on Anderson localization of surface gravity waves
Source: Nat Commun. 2024 Jul 8;15:5726. doi: 10.1038/s41467-024-49575-5 (PMC11231258; doi:10.1038/s41467-024-49575-5)
Supplement: Supplementary file 3 — Description of Additional Supplementary Files [file 41467_2024_49575_MOESM3_ESM.docx]

**Supplementary video legends**

File Name: Supplementary Video 1

Description: Video showing gravity surface waves halted by the periodic bathymetry in a 4-m long canal. 1 Hz forcing. Fluid depth: 5.5 cm. The first 1.2 m of the canal is visible. (11 s, 1.7 Mo).

File Name: Supplementary Video 2

Description: Video showing the spatiotemporal evolution of the surface elevation η(x,t) for a weak forcing (ε ≈ 0.015) and different forcing frequencies f=0.5 Hz (left column), 1 Hz (middle column), and 1.5 Hz (right column) for 7.2 s for a flat bottom (top row), periodic bathymetry (middle row), and random bathymetry (bottom row). Black rectangles indicate the location of bars in the canal. (15 s, 3.9 Mo).

File Name: Supplementary Video 3

Description: Video showing the spatiotemporal evolution of the surface elevation η(x,t) for a strong forcing (ε ≈ 0.040) and different forcing frequencies f=0.5 Hz (left column), 1 Hz (middle column), and 1.5 Hz (right column) for 7.2 s for a flat bottom (top row), periodic bathymetry (middle row), and random bathymetry (bottom row). Black rectangles indicate the location of bars in the canal. (15 s, 2.4 Mo).
