# Supplementary material for: Effects of nonlinearity on Anderson localization of surface gravity waves
Source: Nat Commun. 2024 Jul 8;15:5726. doi: 10.1038/s41467-024-49575-5 (PMC11231258; doi:10.1038/s41467-024-49575-5)
Supplement: Supplementary file 2 — Peer Review File [file 41467_2024_49575_MOESM2_ESM.pdf]

nature portfolio

Peer Review File

Effects of nonlinearity on Anderson localization of surface gravity waves

Open Access This file is licensed under a Creative Commons Attribution 4.0 International License, which permits use, sharing, adaptation, distribution and reproduction in any medium or format, as long as you give appropriate credit to the original author(s) and the source, provide a link to the Creative Commons license, and indicate if changes were made. In the cases where the authors are anonymous, such as is the case for the reports of anonymous peer reviewers, author attribution should be to 'Anonymous Referee' followed by a clear attribution to the source work. The images or other third party material in this file are included in the article's Creative Commons license, unless indicated otherwise in a credit line to the material. If material is not included in the article's Creative Commons license and your intended use is not permitted by statutory regulation or exceeds the permitted use, you will need to obtain permission directly from the copyright holder. To view a copy of this license, visit http://creativecommons.org/licenses/by/4.0/.

REVIEWER COMMENTS

Reviewer #1 (Remarks to the Author):

The authors present intriguing findings regarding the interaction between surface gravity waves and bathymetry of varying types: regular, periodic, and random. Waves of different frequencies are generated in a wave flume (4 m x 18 cm x 5.5 cm), and the surface elevation is monitored using five cameras positioned at different distances along the flume. By utilizing these cameras, the authors can spatially and temporally measure the surface elevation, enabling them to reconstruct the (k-\omega) spectrum. This spectrum is a crucial tool for assessing potential wave excitations. After measuring amplitudes along the channel, the authors observe how wave amplitudes diminish due to multiple resonances. They introduce a localization parameter by fitting an exponential function to the maximum surface elevation along the flume. In addition to altering frequencies, the authors also vary amplitudes and explore the role of nonlinearity in these multi-reflections. They argue that nonlinearity enhances localization; moreover, they claiming to be the first to observe the macroscopic analog of Bloch's dispersion relation.

The experiments are conducted meticulously, and the results appear accurate and reliable. The manuscript is clear and well-written, and I comprehend the authors' interesting findings. However, there appears to be a significant confusion regarding the concept of Anderson localization. The paper's title is "Anderson localization of nonlinear surface gravity waves," yet it seems to me that the manuscript lacks results connected to such a phenomenon. The localization effect observed starting from the beginning of their device occurs for both regular and randomly spaced steps. This suggests that the issue is unrelated to Anderson localization. In disordered media, the Fourier basis becomes inadequate, and it can be demonstrated at a linear level that the eigenfunctions are localized within the media, not at the boundary of discontinuity between regular dynamics and (periodic or) random media. In other words, localization should be observed at some point over the steps and not in the transition region. If the authors disagree with this statement, they should provide an explanation as to why localization is observed in both regular and random bathymetry.

Considering that, in my view, the results are not associated with Anderson localization but rather with Bragg scattering from regular and irregular seabeds, I find the novelty of the manuscript insufficient for acceptance in Nat. Comm. However, if I overlooked any pertinent details in the discussion, I would be open to revising my opinion.

Reviewer #2 (Remarks to the Author):

The authors performed the experiments on surface waves of shallow water, in which the surface elevation was measured by the regularly spaced cameras. The spatiotemporal spectra of surface water waves were calculated and presented for the linear and nonlinear waves. The attenuation and localization of surface waves were observed in the canals with periodic and random bottoms, and the localization length were calculated for different nonlinearities. The authors claimed that the nonlinearity strengthens the localization. However, there are some issues that should be addressed.

1. The authors have only quantified the nonlinearity by the wave steepness, but the details of controlling nonlinearities in the experiments are still missing. Some comments on the specific parameters of the wave maker would be more helpful.

2. Since the full space-and-time-resolved wavefield measurement was employed, is it convenient to provide more measured wave forms in different nonlinearities? The “wave-breaking” results are also expected even in the supplements.

3. The random bottom was realized by moving some bars in the manuscript. How does the only one structure support their claims? What about the other random structures?

4. The surface waves over a length of about 2.5 m were presented. How to choose the starting point of the x-axis? Why?

5. How to measure or calculate the wave amplitude maxima along the canal? Only the wave amplitude maxima in the case of a periodic bottom were presented in Fig. 3. What about these over a random bottom?

6. The nonlinearity was not given in Fig. 3. The wave motion at different nonlinearities is expected.

7. In Fig. 4, the localization length is almost unchanged at 1.1 Hz and decreases more significantly in the case of the periodic bottom than the random bottom. This is somewhat inconsistent with the conclusion. Some detailed comments should be added.

8. The localization length has been derived theoretically, but it is independent of the defined nonlinearity. Can the authors give a theoretical localization length related to the nonlinearity? It can be included in Fig. 4 to compare with the experimental results.

9. The surface tension of water can also cause wave attenuation, which can lead to the similar effects. How to experimentally reduce and theoretically estimate these impacts? Some comments on the surface tension are expected.

10. The significant digits of the same physical quantity should be consistent. Pay attention to the physical unit when numbers and variables are used together.

Reviewer #3 (Remarks to the Author):

The article " Anderson localization of nonlinear surface gravity waves" by G. Ricard et al. presents a 1D water wave-based experiment in which Anderson localization is obtained from a controlled disordered bathymetry. This experiment enables to visualize the evolution of the wave field in space and time with high precision and to extract the full spatiotemporal spectra of the wavefield. The authors show that the non-linear effects strengthen the localization and study this dependence.

This article is quite interesting. It is clearly written and easy to read.

This is an interesting development and extension of the seminal paper of the paper “Localization of Surface Waves on a Rough Bottom: Theories and Experiments” by Belzons et al EPL 1987 with the addition of the effect of non-linearities and a complementary spectral approach. The experimental set-up is trully impressive and reaches a new level that allows the authors to revisit and give new insight into this localization phenomenon that extends to the whole transdisciplinary wave community.

I think this article deserves to be published in Nature Com. I have a few comments:

1. In 1D there is no transition to Anderson localization and thus localization always appear with the presence of disorder (whatever the “strength” of the disorder). It is unfortunately a smooth evolution from the periodic Bragg bathymetry which leads to band gaps to disordered bathymetry leading to disordered-driven localization. Hence, I would like some discussion on the disorder. In particular, how far is the random bathymetry used from the periodic bathymetry in the channel for the waves with a limited length for the canal, for the spectral range used and in the presence of the wave damping? What is the extent of the periodic band gap reminiscence?

2. The addition of NL effects in this 1D configuration makes it even more tricky (with the absence of 1D transition) to relate the induced localization to the so-called Anderson localization (which is a linear effect). I would be more cautious about the relation (in the title and in the text) between the observed 1D localization of NL waves and the term “Anderson localization”… I would at least add a paragraph discussing about it.

3. Reading the manuscript, one is inclined to think of rogue waves and the debated role of non-linearity in this case. Could the authors discuss the link with rogue waves?

Reviewer #1:

The authors present intriguing findings regarding the interaction between surface gravity waves and bathymetry of varying types: regular, periodic, and random. Waves of different frequencies are generated in a wave flume (4 m x 18 cm x 5.5 cm), and the surface elevation is monitored using five cameras positioned at different distances along the flume. By utilizing these cameras, the authors can spatially and temporally measure the surface elevation, enabling them to reconstruct the (k-\omega) spectrum. This spectrum is a crucial tool for assessing potential wave excitations. After measuring amplitudes along the channel, the authors observe how wave amplitudes diminish due to multiple resonances. They introduce a localization parameter by fitting an exponential function to the maximum surface elevation along the flume. In addition to altering frequencies, the authors also vary amplitudes and explore the role of nonlinearity in these multi-reflections. They argue that nonlinearity enhances localization; moreover, they claiming to be the first to observe the macroscopic analog of Bloch's dispersion relation.

⇒ We thank the referee for their remarks and the time spent on the manuscript.

The experiments are conducted meticulously, and the results appear accurate and reliable. The manuscript is clear and well-written, and I comprehend the authors' interesting findings. However, there appears to be a significant confusion regarding the concept of Anderson localization. The paper's title is "Anderson localization of nonlinear surface gravity waves," yet it seems to me that the manuscript lacks results connected to such a phenomenon. The localization effect observed starting from the beginning of their device occurs for both regular and randomly spaced steps. This suggests that the issue is unrelated to Anderson localization

⇒ It is well known that Anderson localization in 1D happens for arbitrarily small disorder. This means that no critical transition exists in 1D from a Bragg scattering regime (periodic case) to a regime of Anderson localization (random case) when the level of disorder is increased. Indeed, in 1D, all eigenstates are localized for arbitrarily small disorder. The principal difference between Bragg scattering and Anderson localization is that, for a periodic lattice it is only the states within the band gap that are localized, while for Anderson all eigenstates are localized. This is also what we observe experimentally.

We agree with the Referee that, in the periodic case, a finite value of the localization length is measured outside the band gap in our experiment. This is simply attributed to the fact that our waves are not infinitesimal as demonstrated by the corresponding numerical simulations in Fig. 4a (blue line) of the manuscript. Furthermore, within the band gap itself, the localization length is found to be significantly smaller than outside, as expected.

Moreover, when the experimental data of the periodic and random cases are superimposed, as in the figure below, we see that the localization length for the random case is always smaller than the ones of the periodic case for small finite amplitude waves. This shows that all the eigenstates are localized, and not simply those within the band gap, as expected for Anderson localization. This is why the comparison to the periodic case is necessary and was introduced in the manuscript. We have added a sentence in the text to clarify this point (lines 336-338).

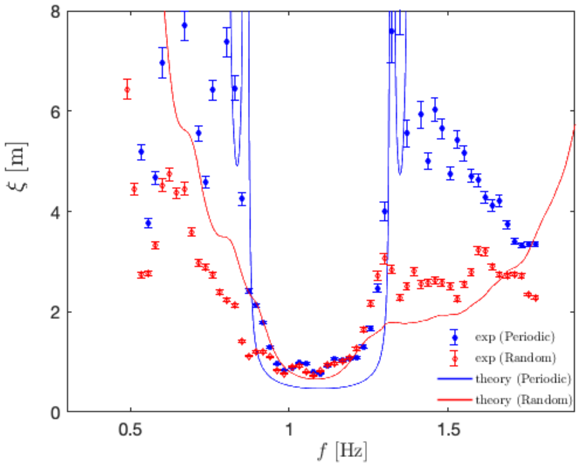

Linked to the Referee's remark, we have also added a discussion (lines 328-346) and a new figure (Fig. 5a) in the manuscript on the effects of the level of disorder on the localization phenomenon. Here, the disorder level is quantified by κ related to the width of the random distribution of distances between bars (see text). Experimentally, we focused on the two extreme cases in which the disorder is 0 (periodic case) and 1 (completely random case) to get the maximal impact of the randomness. We have added the new Figure 5a in the manuscript to show how the localization length ξ is expected to change when the disorder level κ is continuously changed from 0 to 1. Indeed, we observe in Fig. 5a a smooth transition between Bragg scattering (periodic case $\kappa = 0$) to Anderson localization (random cases $\kappa > 0.5$) when the disorder level is increased. We have found that the larger the level of randomness is, the wider the $\xi(f)$ curve is (as all eigenstates are localized and not simply those within the band gap), as also reported for 1D acoustics waves [19, 20]. Only the minimum of the $\xi(f)$ curve is reminiscent of the band gap. This thus confirms that our experimental results for $\kappa = 1$ (completely random bottom) are well ascribed to disorder-driven Anderson localization.

In disordered media, the Fourier basis becomes inadequate, and it can be demonstrated at a linear level that the eigenfunctions are localized within the media, not at the boundary of discontinuity between regular dynamics and (periodic or) random media. In other words, localization should be observed at some point over the steps and not in the transition region. If the authors disagree with this statement, they should provide an explanation as to why localization is observed in both regular and random bathymetry.

⇒ Concerning where and how the localization is observed over our bathymetry, it is unclear what the reviewer means by "localization should be observed at some point over the steps and not in the transition region" since, what we measure are indeed waves above the lattice, and not in a "transition region". All of the measurements of the amplitudes and their exponential decreases are measured above the lattice itself, and not before. It is therefore not some sort of Bragg scattering, which by definition is an effect observed as a reflection of a periodic lattice, and measured outside of the lattice. In our experiment, we measure the wavefield within the lattice.

It seems from the reviewer's comments that for them Anderson localization concerns just the eigenstates, and for that, one should excite one of the eigenfunctions *within* the lattice and show that is localized. To better convince the Referee, one can always consider the following: let some wave arrive onto a random lattice, and some of it is transmitted into the lattice where it undergoes multiple reflections over the randomly placed scatterers. If the eigenfunctions of the system are known, we can write down the wave as a linear superposition of these eigenfunctions and since all eigenstates are localized, the wave within the lattice has to be localized as well. Furthermore, Anderson localization has been experimentally confirmed through the use of this fact in photonics [A], acoustics [21], plasmonics [B], microwaves [C], etc.

Considering that, in my view, the results are not associated with Anderson localization but rather with Bragg scattering from regular and irregular seabeds, I find the novelty of the manuscript insufficient for acceptance in Nat. Comm. However, if I overlooked any pertinent details in the discussion, I would be open to revising my opinion.

⇒ With all the above elements in mind, namely: all states in the random case are found to be localized (with respect to the periodic lattice) as well as the fact that a 1D propagating wave in a random lattice is necessarily localized since it can be decomposed into the eigenstates, we are left to conclude that indeed we have observed Anderson localization of surface gravity waves over a random bathymetry that goes beyond the indirect measurements, obtained forty years ago, for linear surface gravity waves [24, 25].

[A] A. Dikopoltsev, S. Weidemann, M. Kremer, A. Steinfurth, H. H. Sheinfux, A. Szameit, and M. Segev, Observation of Anderson localization beyond the spectrum of the disorder, *Sci. Adv.* 8, eabn7769 (2022).

[B] S. Pandey, B. Gupta, S. Mujumdar, et al., Direct observation of Anderson localization in plasmonic terahertz devices, *Light Sci. Appl.* **6**, e16232 (2017).

[C] A. A. Fernandez-Marin, J. A. Mendez-Bermudez, J. Carbonell, F. Cervera, J. Sanchez-Dehesa, and V. A. Gopar, Observation of Anderson localization beyond the spectrum of the disorder, *Phys. Rev. Lett.* **113**, 233901 (2014).

Additional changes:

- The title of the manuscript has been changed.
- In the experimental setup section, details on the wavemaker motion have been given to better explain how to experimentally increase nonlinear effects.
- The origin $x = 0$ has been moved to the beginning of the lattice, for the sake of clarity.
- To avoid confusion, the theoretical Bloch dispersion relation has been removed from Fig. 2e, 2f as it is theoretically no longer valid for the random case.
- Wave amplitude maxima $\eta_{max}(x)$ along the canal have been added for the random case in Fig. 3b and discussed in the text.
- Typical waveforms for a high nonlinearity ($\epsilon \sim 0.040$) have been added to the Supplemental Material (Section S2) as well as the corresponding Video 3.
- Figure 4 has been enlarged for easier viewing.
- A new Fig. 5b and the corresponding discussion (lines 347-357) have been added to discuss possible finite-size effects of the system on the localization length.
- We have separated multiple references so that a citation lists only one publication as required by Nature Communications policies.
- Previous Extended Data has been included in the Supplemental Material as required by Nature Communications policies.

Reviewer #2:

The authors performed the experiments on surface waves of shallow water, in which the surface elevation was measured by the regularly spaced cameras. The spatiotemporal spectra of surface water waves were calculated and presented for the linear and nonlinear waves. The attenuation and localization of surface waves were observed in the canals with periodic and random bottoms, and the localization length were calculated for different nonlinearities. The authors claimed that the nonlinearity strengthens the localization. However, there are some issues that should be addressed.

⇒ We thank the referee for their report and the time spent on the manuscript. We considered their comments which have improved the quality of the paper.

1. The authors have only quantified the nonlinearity by the wave steepness, but the details of controlling nonlinearities in the experiments are still missing. Some comments on the specific parameters of the wave maker would be more helpful.

⇒ For surface gravity waves, nonlinearity is indeed linked to the wave steepness as $\epsilon \sim \eta_m/\lambda \sim \eta_m f^2$ in the deep-water regime (flat bottom case). Nonlinearity is thus proportional to the rms wave amplitude η_m and the square of its frequency f^2. To experimentally increase nonlinear effects at a fixed frequency, we increase the wavemaker amplitude A. The acceleration range of the wavemaker used is $4 \leq Af^2 \leq 30$ mm/s^2, that corresponds to amplitudes $A \in [1.2, 130.2]$ mm. A sentence has been added to the manuscript on lines 152-154.

2. Since the full space-and-time-resolved wavefield measurement was employed, is it convenient to provide more measured wave forms in different nonlinearities? The "wave-breaking" results are also expected even in the supplements.

⇒ We followed the Referee's suggestion. Typical waveforms for two different nonlinearities ($\epsilon \sim 0.015$, 0.040) are now presented in the revised manuscript: we have added to the Supplemental Material (Sect S2) the waveforms at $\epsilon \sim 0.04$, as well as the corresponding Video 3. No wave breaking is observed experimentally since the nonlinearity is not significant enough.

3. The random bottom was realized by moving some bars in the manuscript. How does the only one structure support their claims? What about the other random structures?

⇒ We thank the Referee for this remark. The disorder level is quantified by κ related to the width of the random distribution of distances between bars (see text). Experimentally, we focused on the two extreme cases in which the disorder is 0 (periodic case) and 1 (completely random case) to get the maximal impact of the randomness. We have added a new Figure 5a in the manuscript to show how the localization length ξ is expected to change when the disorder level κ is continuously changed from 0 to 1. Indeed, we observe in Fig. 5a a smooth transition between Bragg scattering (periodic case $\kappa = 0$) to Anderson localization (random cases $\kappa > 0.5$) when the disorder level is increased. We have found that the larger the level of randomness is, the wider the $\xi(f)$ curve is, as also reported for 1D acoustics waves [19,20]. Only the minimum of the $\xi(f)$ curve is reminiscent of the band gap. This thus confirms that our experimental results for $\kappa = 1$ (completely random bottom) are well ascribed to disorder-driven Anderson localization. Accordingly, we added a discussion (lines 328-346) and the new figure (Fig. 5a) on the effects of the level of disorder on the localization phenomenon.

Note that other types of disorder could also be achieved using steps with random widths or heights. However, only changing the space between successive steps was chosen because it is the easiest and most efficient way to introduce randomness [25]. A sentence has been added to the manuscript (lines 100-103).

4. The surface waves over a length of about 2.5 m were presented. How to choose the starting point of the x-axis? Why?

⇒ The starting point was arbitrarily chosen to allow enough space for the waves to be well-formed after the wavemaker and before the lattice. In the revised version, we have defined the abscissa $x = 0$ when the lattice starts, for the sake of clarity.

5. How to measure or calculate the wave amplitude maxima along the canal? Only the wave amplitude maxima in the case of a periodic bottom were presented in Fig. 3. What about these over a random bottom?

⇒ The wave amplitude maximum η_{max} is measured as the maximal value of η over time at every location x. The figure of $\eta_{max}(x)$ in the random case has been added as Fig. 3b and discussed on lines 250-253.

6. The nonlinearity was not given in Fig. 3. The wave motion at different nonlinearities is expected.

⇒ This typo has been corrected in Fig. 3. New waveforms at different nonlinearities have been added in the Supplemental Material (Sect. S2, Figs. S2 and S3, and Video 3) (see answer to question #2).

7. In Fig. 4, the localization length is almost unchanged at 1.1 Hz and decreases more significantly in the case of the periodic bottom than the random bottom. This is somewhat inconsistent with the conclusion. Some detailed comments should be added.

⇒ We agree that nonlinearity increases wave localization outside of the band gap but persists inside the band gap. We corrected the sentence in the conclusion to make it clearer that the localization length does not change with nonlinearity and keeps its linear value within the band gap (lines 367-369).

8. The localization length has been derived theoretically, but it is independent of the defined nonlinearity. Can the authors give a theoretical localization length related to the nonlinearity? It can be included in Fig. 4 to compare with the experimental results.

⇒ We agree that the localization phenomenon has been derived in the linear regime. A theory that takes into account nonlinear effects would be indeed of paramount importance. To our knowledge, such a theory has not been derived so far and is beyond the scope of the present study. A sentence has been added in the conclusion to comment on this point (lines 381-383).

9. The surface tension of water can also cause wave attenuation, which can lead to the similar effects. How to experimentally reduce and theoretically estimate these impacts? Some comments on the surface tension are expected.

⇒ Capillary and gravity waves are two types of hydrodynamics surface waves with a linear dispersion relation as $\omega^2 = gk + \gamma/\rho k^3$ where γ is the surface tension. The crossover frequency between the two contributions is given by $f_{gc} = \rho^{\frac{1}{4}}\gamma^{-\frac{1}{4}}g^{\frac{3}{4}}/(\sqrt{2}\pi) \approx 14$ Hz which is much larger than the frequencies used in the present study, meaning that surface tension is negligible. A sentence has been added to the manuscript (lines 120-122) to clarify this point.

10. The significant digits of the same physical quantity should be consistent. Pay attention to the physical unit when numbers and variables are used together.

⇒ We thank the Referee for pointing out these typos. We have corrected them throughout the manuscript.

Additional changes:

- The title of the manuscript has been changed.
- To avoid confusion, the theoretical Bloch dispersion relation has been removed from Fig. 2e and 2f as it is theoretically no longer valid for the random case.
- Figure 4 has been enlarged for easier viewing.
- Figure 5, as well as the corresponding discussion, has been added (lines 328-357).
- We have separated multiple references so that a citation lists only one publication as required by Nature Communications policies.
- Previous Extended Data has been included in the Supplemental Material as required by Nature Communications policies.

Reviewer #3:

The article " Anderson localization of nonlinear surface gravity waves" by G. Ricard et al. presents a 1D water wave-based experiment in which Anderson localization is obtained from a controlled disordered bathymetry. This experiment enables to visualize the evolution of the wave field in space and time with high precision and to extract the full spatiotemporal spectra of the wavefield. The authors show that the non-linear effects strengthen the localization and study this dependence.

This article is quite interesting. It is clearly written and easy to read.

This is an interesting development and extension of the seminal paper of the paper “Localization of Surface Waves on a Rough Bottom: Theories and Experiments” by Belzons et al EPL 1987 with the addition of the effect of non-linearities and a complementary spectral approach. The experimental set-up is trully impressive and reaches a new level that allows the authors to revisit and give new insight into this localization phenomenon that extends to the whole transdisciplinary wave community.

I think this article deserves to be published in Nature Com. I have a few comments:

⇒ We thank the referee for their positive report and the time spent on the manuscript. We considered their comments which have improved the quality of the paper.

1. In 1D there is no transition to Anderson localization and thus localization always appear with the presence of disorder (whatever the “strength” of the disorder). It is unfortunately a smooth evolution from the periodic Bragg bathymetry which leads to band gaps to disordered bathymetry leading to disordered-driven localization. Hence, I would like some discussion on the disorder. In particular, how far is the random bathymetry used from the periodic bathymetry in the channel for the waves with a limited length for the canal, for the spectral range used and in the presence of the wave damping? What is the extent of the periodic band gap reminiscence?

⇒ We followed the Referee’s suggestion and added a discussion (lines 328-346) and a new figure (Fig. 5a) on the effects of the level of disorder on the localization phenomenon. Here, the disorder level is quantified by κ related to the width of the random distribution of distances between bars (see text). Experimentally, we focused on the two extreme cases in which the disorder is 0 (periodic case) and 1 (completely random case) to get the maximal impact of the randomness. We have added the new Figure 5a in the manuscript to show how the localization length ξ is expected to change when the disorder level κ is continuously changed from 0 to 1. Indeed, we observe in Fig. 5a a smooth transition between Bragg scattering (periodic case $\kappa = 0$) to Anderson localization (random cases $\kappa > 0.5$) when the disorder level is increased. We have found that the larger the level of randomness is, the wider the $\xi(f)$ curve is, as also reported for 1D acoustics waves [19,20]. Only the minimum of the $\xi(f)$ curve is reminiscent of the band gap. This thus confirms that our experimental results for $\kappa = 1$ (completely random bottom) are well ascribed to disorder-driven Anderson localization.

We have also added Fig. 5b and the corresponding discussion (lines 347-357) on the possible finite-size effects of the system on the localization length. We have concluded that the experiments are only slightly affected by finite-size effects.

Concerning the possible wave damping by viscosity, as explained in Section 5 of the Supplemental Material (see also Fig. S5 of the Supp. Mat), the theoretical dissipation length is much larger than the canal length and agrees with the experimental estimation ~ 20 m.

2. The addition of NL effects in this 1D configuration makes it even more tricky (with the absence of 1D transition) to relate the induced localization to the so-called Anderson localization (which is a linear

effect). I would be more cautious about the relation (in the title and in the text) between the observed 1D localization of NL waves and the term “Anderson localization”… I would at least add a paragraph discussing about it.

⇒ We agree with the Referee that Anderson localization is a linear effect and, indeed, talking about Anderson localization of nonlinear waves can be misleading. Accordingly, the title of the manuscript has been changed (“Effects of nonlinearity on Anderson localization of surface gravity waves”, which is more accurate) as well as sentences in the introduction (lines 39-41), the main part (lines 309-313) and the conclusion (lines 381-383).

3. Reading the manuscript, one is inclined to think of rogue waves and the debated role of nonlinearity in this case. Could the authors discuss the link with rogue waves?

⇒ We thank the Referee for suggesting this possible extension of our work. We added a sentence at the end of the conclusion.

Additional changes:

- In the experimental setup section, details on the wavemaker motion have been given to better explain how to experimentally increase nonlinear effects.
- The origin $x = 0$ has been moved to the beginning of the lattice, for the sake of clarity.
- Wave amplitude maxima $\eta_{max}(x)$ along the canal have been added for the random case in Fig. 3b and discussed in the text.
- Typical waveforms for a high nonlinearity ($\epsilon \sim 0.040$) have been added to the Supplemental Material (Section S2) as well as the corresponding Video 3.
- To avoid confusion, the theoretical Bloch dispersion relation has been removed from Fig. 2e and 2f as it is theoretically no longer valid for the random case.
- Figure 4 has been enlarged for easier viewing.
- We have separated multiple references so that a citation lists only one publication as required by Nature Communications policies.
- Previous Extended Data has been included in the Supplemental Material as required by Nature Communications policies.

REVIEWER COMMENTS

Reviewer #1 (Remarks to the Author):

I've read the authors' responses, but unfortunately, they aren't entirely convincing. My main contention is that in both the periodic and random scenarios, the authors observe a reflection in the transition region between the free propagation and the steps. While it's true that the authors measure over the steps, what they're actually measuring is simply an exponential decay of amplitudes starting from the boundary of the steps. A similar decay is noticed in the case of a periodic structure.

What becomes evident from these experiments is that Bragg scattering is less effective when the steps aren't perfectly periodic, but at the same time more frequencies are reflected because the structure contains more scales. If the phenomenon observed were Anderson localization, then by altering the randomness, they should observe localization in a different location along the flume. However, this isn't the case; the maximum amplitude consistently appears in the same place.

I would have no objections to accepting the manuscript if the authors could alter the position of the localization.

Reviewer #2 (Remarks to the Author):

The authors have answered the questions.

Reviewer #3 (Remarks to the Author):

The authors answered to all my remarks and comments. My concerns have been addressed in the revisions.

2nd report of the Reviewer #1:

I've read the authors' responses, but unfortunately, they aren't entirely convincing. My main contention is that in both the periodic and random scenarios, the authors observe a reflection in the transition region between the free propagation and the steps. While it's true that the authors measure over the steps, what they're actually measuring is simply an exponential decay of amplitudes starting from the boundary of the steps. A similar decay is noticed in the case of a periodic structure.

⇨ As already explained in our first response to Reviewer 1 and the figure included in that previous response (figure reproduced below), the exponential decays are found to be **different** experimentally (up to a factor 3) in the periodic (blue) and random (red) bottom case. This figure comparing random and periodic results has been added in the Supp. Information Sect. 4. The origins of the exponential decays are different. In the periodic bottom, a significant exponential decrease is observed experimentally within the bandgap in agreement with Floquet theory and the low exponential decrease outside the bandgap is due to the finite wave amplitude in contrast to the infinitesimal amplitude of the linear theory (see blue line of the figure below) and confirmed by our numerical simulations (see Fig. 4a, blue solid line). We have added a sentence (lines 289-291) to better clarify this point.

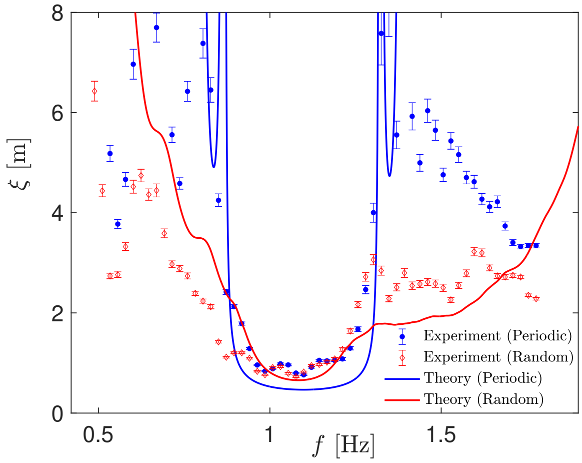

What becomes evident from these experiments is that Bragg scattering is less effective when the steps aren't perfectly periodic, but at the same time more frequencies are reflected because the structure contains more scales.

⇨ Contrary to the statement of the Reviewer, it is clearly demonstrated, experimentally and theoretically, in the figure included in our first response (figure reproduced above), that the effect of the topography is more effective (i.e., waves are more attenuated) as the level of disorder increases (see also Fig. 5a).

If the phenomenon observed were Anderson localization, then by altering the randomness, they should observe localization in a different location along the flume. However, this isn't the case; the maximum amplitude consistently appears in the same place.

I would have no objections to accepting the manuscript if the authors could alter the position of the localization.

⇨ In addition to our above arguments based on experimental, numerical, and theoretical facts, we add remarks below to attempt to resolve Reviewer 1's potential misunderstanding.

First, we have the feeling that Reviewer 1's remark stems from a quantum reasoning based on the motion of particles. Here, we recall that we are performing experiments with classical (hydrodynamic) waves that differ from an electron or a probabilistic wave-particle duality that could propagate and be scattered by several

scattering centers (depending on the degree of disorder) before being localized. We believe that the Reviewer is still misunderstanding that we focus on propagating classical waves that are continuously injected and whose localization properties are different than that of a wavepacket. To our knowledge, for classical monochromatic waves, such as ours, no experiment has demonstrated that the localization position depends on the level of disorder in 1D. Moreover, other experiments with continuous waves suggest that the higher the level of disorder, the stronger the exponential localization of the waves around the source, e.g., in optics [Segev] or acoustics [Ye].

Moreover, the incidence of the waves from the right, the left, the middle, or what the Reviewer calls, "free propagation region", does not influence localization. Indeed, the exponential decrease would be the same if the lattice extended further to the other side of the wavemaker, and hence it would be positioned in the middle of a disordered lattice. The wavemaker would then act as a source of a wave in the middle of this lattice, which would be localized around the source. These two pictures are physically equivalent with the same phenomenology. The exponential decay of transmission of a wave, and in particular a classical wave, is well known and established in the literature, and our experiments align with these predictions [Asatryan]. Not only that, even in the case of wavepackets, their exponential localization is observed **around the point of injection**, for example with light [Dikopoltsev].

Furthermore, the exponential decay of the transmission in a disordered medium is a well-known signature of Anderson localization as demonstrated in countless examples and experiments. In localization of light, this fact is stressed in [Wiersma], stating exponential decay of the intensity around a source positioned in the middle of the lattice, as well as exponential decay upon entering from the outside. The same exponential decay is observed and modeled in many cases of transverse localization of waves in disordered arrays and is observed around a source that injects energy into the lattice. This is due to Anderson localization, both in the case of light [Segev] as well as sound waves [Ye].

Furthermore, Fig. 5a theoretically shows that the exponential decay of the wave, starting from its emission, becomes stronger as the degree of disorder increases continuously. This is confirmed experimentally. In the random case, the only reminiscence of Bragg scattering is the minimum of the \xi(f) curve. A factor of up to 3 times is shown experimentally for the \xi value between the periodic and random cases - see figure above). **Our experimental observations follow qualitatively and quantitatively predictions coming from the theory of Anderson localization in 1D** (as done in references already presented in our previous reply). This confirms that our experimental results, in the completely disordered case, are indeed ascribed to disorder-driven Anderson localization.

As localization of classical waves has already been published in many fields (optics, hydrodynamics, microwaves, acoustics, photonics, plasmonics), mostly demonstrated by experimental measurements of transmission and reflection, after and before the grating (and not on the whole disordered grating as here), we do not understand the Reviewer's position, which implicitly doubts all these articles published in these different fields. We hope these clarifications will enable Reviewer 1 to find this manuscript worthy of publication, as the other two Reviewers did.

Bibliography

M. Segev, Y. Silberberg, and D. Christodoulides, Anderson localization of light. Nature Photon. **7**, 197 (2013).

Y. Ye et al.,Transversal Anderson localization of sound in acoustic waveguide arrays**,** J. Phys. Cond. Matter **27,** 155402 (2015).

A. Asatryan et al., Anderson localization of classical waves in weakly scattering metamaterials, Phys. Rev. B **81**, 075124 (2010).

A. Dikopoltsev et al., Observation of Anderson localization beyond the spectrum of the disorder, Sci. Adv. **8**, eabn7769 (2022).

D. Wiersma et al., Localization of light in a disordered medium, Nature **390**, 671 (1997).

REVIEWERS' COMMENTS

Reviewer #1 (Remarks to the Author):

Although I'm not entirely convinced that the authors are indeed observing Anderson localization, I believe the manuscript is interesting and that the results merit publication. The scientific community and future research will surely shed more light on this aspect.

We would like to thank the reviewer #1 for the comments. As reviewer #1 suggested, we have included the R code as a supplement to the manuscript, specific and detailed methods for the related analysis are also provided with code. Here, we present a comprehensive point-by-point response to address each of the reviewers' comments (underlined):

Reviewer #1 (Remarks to the Author):

I appreciate the authors' efforts to address my concerns, and I admit that they are specialized to the mass spectrometry proteomics.

However, I'm disappointed to note that my two points remain unsatisfactorily addressed.

(1) The additional methods detail is helpful, but simply using the limma R package doesn't inherently deal with the uneven replication. While limma does not require equal variances, specific parameters must be used, e.g. to denote weights, for limma to allow different variabilities for the treatment groups. Perhaps sharing as a supplemental file the R code used to generate the volcano plot, showing how to filter the Proteome Discover data and perform the statistical testing would be helpful to share (especially for transparency and reproducibility)?
Response: Thank you for your comments. As you suggested, we have included the R code with details as a supplement to the manuscript (described in Lines 411-421), and also discussed the technical limitations of the analysis in the discussion section (Lines 330-336).

(2) Raw data (in this edit, the identifier PXD050294) is not findable in the respository.
Response: We have uploaded the raw data of mass in iProx on March 4th, 2024 with the access level as public after acceptance (means that we will open it to public when this MS is accepted). We apologize for the inconvenience due to our caution and unfamiliar with this system. Please verify this data using the provided link (https://www.iprox.cn/page/PSV023.html;?url=1714711122768IU1s) and key (Fpco), and we will update its access level to public upon our work is accepted. Actually, we would like to update its access level now, but we are afraid of causing some change when we update it, resulting in further misunderstanding.

#####
Additional comments:
Overall, the code does clarify the analysis (I'd highly recommend the code be shared as supplemental information!), and my main concern about biological replication and data massaging influencing the biological conclusions -- that is presumably address by the other reviewers' expertise looking at the follow-up

experiments to validate the mass spectrometry results, and I defer to their judgement on those sections of the paper!

Response: Thanks for your comments. As suggested, we have included the R code as a supplement to the manuscript. Indeed, biological replication and data massaging may influence the conclusions. However, based on valuable insights provided by mass data, subsequent biological experiments further supported the important role of RPLP1 in regulating HIV-1, which is the major part of our work. Taken the analysis of mass data and lots of biological experiments together, we concluded that RPLP1 inhibits HIV-1 replication by occupying the C/EBPβ binding sites in the viral LTR.
